# What's the Risk? Older Women Report Fewer Symptoms for Suspected Acute Coronary Syndrome than Younger Women

**DOI:** 10.1089/biores.2018.0020

**Published:** 2018-09-18

**Authors:** Holli A. DeVon, Karen Vuckovic, Larisa A. Burke, Sahereh Mirzaei, Katherine Breen, Nadia Robinson, Jessica Zegre-Hemsey

**Affiliations:** ^1^ Department of Biobehavioral Health Science, College of Nursing, University of Illinois at Chicago, Chicago, Illinois.; ^2^ School of Nursing, University of North Carolina, Chapel Hill, North Carolina.

**Keywords:** acute coronary syndrome, age, risk, symptoms, women

## Abstract

The purpose of the study was to determine whether older (≥65 years) and younger (<65 years) women presenting to the emergency department (ED) with symptoms suggestive of acute coronary syndrome (ACS) varied on risk factors, comorbid conditions, functional status, and symptoms that have implications for emergent cardiac care. Women admitted to five EDs were enrolled. The ACS Symptom Checklist was used to measure symptoms. Comorbid conditions and functional status were measured with the Charlson Comorbidity Index and Duke Activity Status Index. Logistic regression models were used to evaluate symptom differences in older and younger women adjusting for ACS diagnosis, functional status, body mass index (BMI), and comorbid conditions. Analyses were stratified by age, and interaction of symptom by age was tested. Four hundred women were enrolled. Mean age was 61.3 years (range 21–98). Older women (*n* = 163) were more likely to have hypertension, hypercholesterolemia, never smoked, lower BMI, more comorbid conditions, and lower functional status. Younger women (*n* = 237) were more likely to be members of minority groups, be college-educated, and have a non-ACS discharge diagnosis. Younger women had higher odds of experiencing chest discomfort, chest pain, chest pressure, shortness of breath, nausea, sweating, and palpitations. Lack of chest symptoms and shortness of breath (key symptoms triggering a decision to seek emergency care) may cause older women to delay seeking treatment, placing them at risk for poorer outcomes. Younger African American women may require more comprehensive risk reduction strategies and symptom management.

## Introduction

Despite advances in medical care and treatment, the burden of heart disease in the United States remains high, and women bear a disproportionate amount.^1^ Heart disease claims more women's lives than all forms of cancer combined and is responsible for one in three deaths among women.^1^ Despite these troubling statistics, many women believe that heart disease is a man's disease and underestimate their own risk. In 2013, Mosca reported that only 57% of women were aware that heart disease is their primary health threat.^2^ The level of heart disease awareness among African American and Hispanic women was 36% and 34%, respectively (similar to the level of white women in 1997).^2^ Women aged 55 years or less comprise almost one-fifth of all cases of acute coronary syndrome (ACS).^3^ Paradoxically, younger women have higher mortality rates from ACS than age-matched men and older women,^4^ which Izadnegahdar et al. suggested is likely related to worsening cardiac risk factors, including obesity, smoking, hypertension, and diabetes. Recently, Hayes et al. reported that spontaneous coronary artery dissection may account for up to 35% of myocardial infarctions (MIs) in women ≤50 years.^5^

After age 65, the risk of heart disease in women is equivalent to men, with women experiencing MI at an average age of 71.7 years.^6^ Risk factors unique to older women include menopause, hormone replacement therapy, and previous treatment for breast cancer.^7,8^ Several risk factors related to pregnancy have been identified as affecting younger women. In a review of associations between preterm delivery and future maternal cardiovascular disease, Minissian et al. suggested that abnormal responses to pregnancy were a woman's first physiological “stress test.”^9^ Veerbeek et al. found that 39% of women with pregnancy-induced hypertension continued to have elevated blood pressure in the postpartum period.^10^ Gestational diabetes has also been linked to an increased lifetime risk of type 2 diabetes (T2DM), a coronary heart disease equivalent. In a study of 2787 women in the Coronary Artery Risk Development in Young Adults Study (CARDIA), a history of gestational diabetes was a marker for early atherosclerosis independent of prepregnancy obesity among women who had not developed T2DM or metabolic syndrome.^11^ These findings suggest that older women who experienced abnormal pregnancies earlier in life may be at added risk for ACS.

Younger women are in danger of failing to recognize ACS symptoms and reacting rapidly.^12^ Prior studies demonstrated that younger women experience prodromal symptoms more often than older women before an episode of ACS.^13,14^ Younger women also experience a more diverse range of symptoms, from pain or discomfort in the chest, neck, or jaw, to sweating, fatigue, and dizziness; however, chest pain or discomfort remains the most prevalent symptom.^15^ In contrast, women ≥65 years with ACS have been shown to experience fewer symptoms, less chest pain, and more dyspnea than younger women.^16^ Many studies of symptoms of ACS focused on sex differences,^17^ but few focused on how symptoms may vary between older and younger women.^16^ It is important to determine (1) how symptoms experienced by older and younger women vary during an episode of potential ACS, (2) whether differences in symptoms are significant enough to warrant intervention, and (3) what interventions would be appropriate to improve clinical and patient outcomes.

The purpose of this study was to determine whether older (≥65 years) and younger (<65 years) women presenting to the emergency department (ED) with suspected ACS varied on risk factors, comorbid conditions, functional status, and symptoms. “Older” was defined as ≥65 years because women are eligible for Medicare insurance at age 65, prehospital delay times for potential ACS are increased for this group,^18^ and the risk of heart disease reaches parity for women and men at this age.^6^

## Materials and Methods

This analysis is part of a larger prospective, multicenter study examining the influence of gender on symptom characteristics during ACS.^19^ Patients were enrolled at five large referral centers in the Midwest, West, Southwest, and the Pacific Northwest regions of the United States. The centers included four academic medical centers and a large referral community medical center. Approval from all five institutional review boards and the sponsoring institution was received before the start of the study. Each institutional review board approved a waiver of initial consent for electronic screening of patients and to collect initial symptom data before enrollment. A waiver of initial consent was granted because the main study aim was to evaluate symptoms on presentation to the ED and because of the emergent nature of patients presenting with possible ACS. All patients gave written, informed consent before enrollment in the study. Symptom data were destroyed if patients declined to participate.

### Study population

Women and men presenting to the ED between January 2011 and December 2013 were enrolled. Inclusion criteria were symptoms resulting in a cardiac evaluation, fluent in English or Spanish, ≥21 years, and self-presented or were transported by emergency medical services. Patients were excluded if they had an exacerbation of heart failure, were transferred from a hemodialysis unit, were referred for evaluation of a dysrhythmia, or had cognitive impairment. A targeted sampling plan was employed because most patients presenting to the ED for symptoms suggestive of ACS will rule-out. Patients most likely to rule-in were identified by research staff before enrollment, based on electrocardiogram (ECG) and troponin criteria. Research staff approached patients with a troponin level outside the referenced norm for the institution and/or with any ECG changes suggestive of ischemia for enrollment. Ischemia was defined as T-wave inversion, ST-depression, or ST-elevation ≥1 mm in two contiguous leads (except for V2–V3, which have cut points of ≥2 mm for men ≥40 years, ≥1.5 mm for men <40 years, and ≥2.5 mm for women).^20^

### Measures

#### Demographic data

Demographic measures included age, sex, education, income, marital status, and income.

#### ACS patient information questionnaire

This demographic and clinical questionnaire was designed by using the standardized reporting guidelines for studies evaluating risk stratification of ED patients with potential ACS.^21^ The criteria were established by the Multidisciplinary Standardized Reporting Criteria Task Force and are supported by the Society for Academic Emergency Medicine, American College of Emergency Physicians, American Heart Association (AHA), and American College of Cardiology (ACC). The purpose of the document is to establish standardized reporting criteria that will more easily allow for study comparisons and meta-analyses, one of the rationales for the proposed study. The questionnaire takes about 10 min to complete. It was used to generate appropriate descriptive statistics for the sample.

#### The ACS Symptom Checklist

The ACS Symptom Checklist is a 13-item empirically derived instrument that measures the symptoms of ACS. The ACS Symptom Checklist has demonstrated reliability and validity in prior studies.^22,23^ Participants indicate whether the symptom is present or absent. Other symptoms can be recorded in a blank space marked “other.” Each symptom is analyzed individually, and there is no summary score.

#### Charlson Comorbidity Index

The 19-item weighted index is the most extensively studied method of quantifying risk associated with comorbid conditions.^24^ Higher scores represent a greater burden of disease. Studies have demonstrated that the Charlson Comorbidity Index (CCI) is a valid measure for predicting disability and death after ischemic stroke and heart disease,^25^ as well as for in-hospital and 1-year outcomes in patients with ACS.^26^

#### Duke Activity Status Index

The Duke Activity Status Index (DASI) is a brief 12-item instrument that measures functional capacity.^27^ Scores range from 0 to 58.2, with higher scores representing better physical functioning. The items on the scale are weighted to reflect metabolic energy expenditure and correlate highly with peak VO_2_ (r = 0.80, *p* < 0.0001) in patients with ACS^28^ and ischemic heart disease.^29^ Concurrent validity was supported by correlations with measures of physical functioning (r = 0.69, *p* < 0.05 and r = 0.61, *p* < 0.05).^30^

### Procedures

The ACS symptom checklist was completed by a member of the research staff when the patient arrived in ED triage. In most cases, symptoms were assessed within 15 min of presentation. Patients were approached by the research staff for enrollment after they were moved to an ED examination room or to a cardiac bed in the hospital. The study was explained by a member of the research staff, and, once the patient provided written informed consent, additional clinical and individual characteristics were recorded. If the patient declined to participate, the ACS symptom checklist was shredded.

### Statistical analysis

Study data were entered into SPSS (IBM SPSS Statistics for Windows, version 24.0, IBM Corp., Armonk, NY) by a research assistant and transferred to SAS (version 9.3; Cary, NC) for analysis. Significance was set at *p* < 0.05 for all statistical procedures. Normally distributed continuous variables were described with means, and independent-sample *t* tests or analysis of variance were performed. Categorical data were described with frequencies and compared across diagnostic categories with the Chi-square test for independence.

Logistic regression models were used to evaluate symptom differences in older and younger women, adjusting for ACS diagnosis, functional status, body mass index (BMI), and comorbid conditions. Analyses of symptoms were stratified by age, and interaction of symptom by age was tested. Potential covariates were chosen because of reports of gender differences in prior studies^31–33^ and because obesity,^17^ diabetes,^34^ and functional status^35^ can confound the symptom experience.

## Results

### Demographics

The sample (*n* = 400) included 237 women <65 years and 163 women ≥65 years. Mean age was 61.3 years (range 21–98 years). Younger women were more likely to be members of minority groups (36.2% vs. 25.9%), have a higher level of education (*p* = 0.028), and have a non-ACS discharge diagnosis (70.9% vs. 62.0%; Table 1).

**Table 1.** **Demographic and Clinical Characteristics of the Sample**

	<65 Years	≥65 Years	
Characteristic	*n* = 237 (%)	*n* = 163 (%)	*p*
Race/ethnicity [*n* (%)]			0.042
African American	40 (17.2)	21 (13.0)	
White-non Hispanic	150 (64.7)	120 (74.1)	
Hispanic	14 (6.0)	13 (8.0)	
Other	28 (12.1)	8 (4.9)	
Education, *n* (%)			0.028
Less than high school diploma	22 (9.4)	30 (18.4)	
High school diploma	47 (20.1)	41 (25.2)	
Some college	81 (34.6)	45 (27.6)	
College degree/Graduate work	57 (24.4)	28 (17.2)	
Graduate degree	27 (11.5)	19 (11.7)	
Diagnosis			0.048
Non-ACS	165 (71.1)	100 (61.7)	
Unstable angina	20 (8.6)	10 (6.2)	
NSTEMI	33 (14.2)	40 (24.7)	
STEMI	14 (6.0)	12 (7.4)	
BMI [mean (SD)]	31.3 (8.9)	28.5 (6.8)	0.001
Smoking status, *n* (%)			<0.001
Never	133 (58.3)	108 (67.5)	
Former	46 (20.2)	43 (26.9)	
Current smoker	49 (21.5)	9 (5.6)	
Hypertension, *n* (%)	127 (54.7)	117 (73.1)	<0.001
Hypercholesterolemia, *n* (%)	113 (48.9)	103 (64.0)	0.003
CCI Score, mean (SD)	1.4 (1.7)	2.3 (2.0)	<0.001
DASI Score, mean (SD)	34.0 (19.6)	23.1 (16.5)	<0.001

ACS, acute coronary syndrome; BMI, body mass index; CCI, Charlson Comorbidity Index; DASI, Duke Activity Status Index; NSTEMI, non-ST elevation myocardial infarction; SD, standard deviation; STEMI, ST-elevation myocardial infarction.

### Comorbid conditions and functional status

Older women (*n* = 163), compared with younger women, were more likely to have hypertension (*p* < 0.001), hypercholesterolemia (*p* = 0.003), never smoked (*p* < 0.001), a lower BMI (*p* = 0.001), more comorbid conditions (*p* < 0.001), and lower functional status (*p* < 0.001). Younger women (*n* = 237) were more likely to be members of minority groups (*p* = 0.042), be college-educated (*p* = 0.028), and to have a non-ACS discharge diagnosis (0.048). Younger women also had higher odds of experiencing chest discomfort (odds ratio; OR = 2.50, 95% confidence interval; CI = 1.51–4.15), chest pain (OR = 1.65, 95% CI = 1.02–2.65), chest pressure (OR = 2.21, 95% CI = 1.36–3.58), shortness of breath (OR = 2.21, 95% CI = 1.36–3.58), nausea (OR = 1.80, 95% CI = 1.12–2.87), sweating (OR = 1.87, 95% CI = 1.15–3.04), and palpitations (OR = 1.79, 95% CI = 1.08–2.97).

As expected, older women were more likely to have hypercholesterolemia (64% vs. 49.1%) and hypertension (73.1% vs. 54.5%), as well as higher mean scores on the CCI (2.3 ± 2.0 vs. 1.4 ± 1.7). Younger women had higher BMI (31.3 ± 8.9 vs. 28.5 ± 6.8), were more likely to smoke (21.5% vs. 5.6%), and had higher physical functioning as measured by the DASI (34.1 ± 19.5 vs. 23.1 ± 16.5; Table 1).

### Symptom differences

**FIG. 1.**
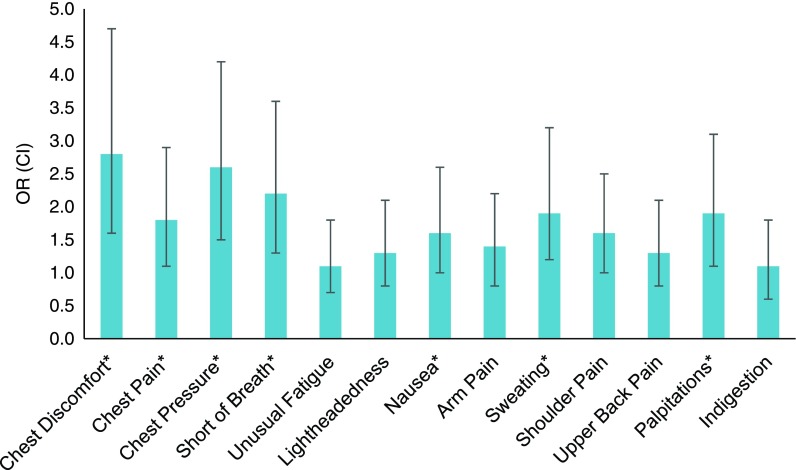
Adjusted odds of reporting a symptom for younger compared with older women. Symptoms are listed in order of most frequently to least frequently reported. Adjusted for acute coronary syndrome diagnosis, Charlson Comorbidity Index, Duke Activity Status Index, and body mass index. *Statistically significant difference.

In unadjusted analyses, older women, compared with younger women, were less likely to report 9 of 13 symptoms: chest discomfort, chest pain, chest pressure, shortness of breath, nausea, arm pain, sweating, shoulder pain, and palpitations (Table 2). After adjusting for diagnosis, CCI, DASI, and BMI, arm and shoulder pain were no longer significantly different between older and younger women. Younger women had higher odds of experiencing chest discomfort (OR = 2.46, 95% CI = 1.48–4.09), chest pain (OR = 1.63, 95% CI = 1.01–2.63), chest pressure (OR = 2.42, 95% CI = 1.48–3.96), shortness of breath (OR = 2.15, 95% CI = 1.32–3.49), nausea (OR = 1.73, 95% CI = 1.08–2.78), sweating (OR = 1.85, 95% CI = 1.13–3.03), and palpitations (OR = 1.78, 95% CI = 1.07–2.97; Fig. 1).

**Table 2.** **Unadjusted Symptom Differences by Age Group**

	<65 Years	≥65 Years	
Symptom^a^	*n* = 237 (%)	*n* = 163 (%)	*p*
Chest discomfort	191 (80.6)	94 (57.7)	<0.001
Chest pain	174 (73.4)	92 (56.4)	<0.001
Chest pressure	181 (76.4)	90 (55.2)	<0.001
Short of breath	160 (67.5)	85 (52.1)	0.002
Unusual fatigue	119 (50.2)	81 (49.7)	0.919
Lightheadedness	126 (53.2)	75 (46.3)	0.178
Nausea	116 (48.9)	60 (36.8)	0.016
Arm pain	103 (43.5)	53 (32.5)	0.027
Sweating	98 (41.4)	44 (27.0)	0.003
Shoulder pain	107 (45.1)	53 (32.5)	0.011
Upper back pain	94 (39.7)	56 (34.4)	0.281
Palpitations	92 (38.8)	38 (23.3)	0.001
Indigestion	67 (28.3)	38 (23.3)	0.268

^a^ Symptoms are listed in order of most frequently to least frequently reported.

In unadjusted analyses, older women reported fewer symptoms than younger women in 9 of 12 symptoms measured. After adjusting for variables that are known to confound the symptom experience (comorbid conditions,^36^ functional status,^35^ and diabetes^37^), the only difference in findings was that arm and shoulder pain were not reported more frequently by younger women. Arm pain was the second most reported symptom, after chest pain, in a cohort of women with spontaneous coronary artery dissection,^38^ and, in another study, women with arm pain had higher odds of an ACS diagnosis compared with men.^39^

## Discussion

Congruent with previous studies,^40^ there were differences in risk factors, comorbid conditions, and symptoms between older and younger women in our study. Key findings included that older women were more likely to have hypertension, hypercholesterolemia, never smoked, lower mean BMI, more comorbid conditions, and poorer functional status. Younger women were more likely to be members of minority groups, be college-educated, and have a non-ACS discharge diagnosis. Younger women had higher odds of experiencing chest discomfort, chest pain, chest pressure, shortness of breath, nausea, sweating, and palpitations after adjusting for covariates.

Risk factors and comorbid conditions may contribute to poorer patient-centered outcomes for both groups of women, including confusion over the cause of symptoms and delay in presentation to the ED. These factors may also contribute to breakdowns in clinical care, such as prolonged time to ECG acquisition, inappropriate cardiac catheterization laboratory activation, or missed ACS diagnosis. As anticipated, we found that older women were more likely to have risk factors, comorbid conditions, and poorer physical functioning compared with younger women. These factors may disadvantage older women who experience symptoms of ACS because they may misinterpret symptoms and attribute them to a nonemergent condition^41^ or to the aging process.^42^ Women may also be unaware of their risk for heart disease^2^ or lack knowledge^43^ of symptoms. Risk factors associated with pregnancy (including pregnancy-induced hypertension,^44^ gestational diabetes,^45,46^ and preterm delivery^47^) are associated with additional risk for future cardiovascular disease; however, it remains unclear as to whether these risks contribute to symptom differences for older versus younger women.

The increased prevalence of comorbidities in women plays a key role in their risk of cardiovascular disease. Prevalence of obesity is on the rise worldwide,^48^ and cardiovascular disease occurs at a younger age in obese women than nonobese women.^49^ Obese women experience more metabolic disorders such as hyperlipidemia, T2DM, and insulin resistance.^49^ Vishram et al. found that the prevalence of metabolic syndrome increased fivefold in women from age 19 to 79.^50^ Obese women also experience greater increases in systolic and diastolic blood pressure compared with women with a normal BMI.^51^ This may partly explain why younger women in our sample were more likely to be members of a minority, given the higher prevalence of obesity in African American and Hispanic women.^52^ Hypertension is another comorbidity adversely affecting women. In 2017, the proportion of women aged 45–54 years with hypertension was estimated to be 33%,^53^ and 62% of Americans with hypertension are female.^54^ The incidence of hypertension in postmenopausal women is four times higher than in premenopausal women.^53^ Finally, although smoking is a well-known and modifiable risk factor, middle-aged women who smoke have a sixfold increased risk of cardiovascular disease compared with a threefold increased risk in men.^54^

Younger women may be at higher risk for a missed ACS diagnosis. Clinicians may have a low suspicion for ACS even in the presence of chest pain and associated symptoms, given the lower likelihood of ACS in younger women.^55^ Previous studies have highlighted significant disparities in acute cardiovascular care among women, minorities, and the elderly.^55^ Younger women are also at higher risk for failing to recognize ACS symptoms and calling emergency medical services,^56^ particularly minority women, given their lack of awareness of their vulnerability to heart disease.^2^ Several heart disease awareness campaigns are attempting to increase women's awareness of risk,^57,58^ but progress is slow and Mosca et al. found that minority women's awareness of their risk of heart disease lags behind that of white women.^2^ Efforts such as these are necessary to equip women with information about this disease and its associated traditional and nontraditional risk factors and symptoms. Younger women in our study were more highly educated, which may have implications for future interventions targeted to both younger and older women. Younger women who are more highly educated and exposed to social media may be more aware of cardiovascular risk factors and their own susceptibility to heart disease. Our data on symptom differences by age may help inform the design of age-appropriate interventions for younger and older women. Younger women can receive reinforcement of their risk for heart disease before they develop ACS. In contrast, older women may have a greater need for information on timely treatment-seeking and symptom recognition.

The fact that the older women in our sample experienced less chest pain, pressure, and discomfort is concerning from the perspective of the patient and clinicians who may suspect alternative and less emergent diagnoses, including gastric reflux or heartburn. Risks of delayed presentation and care are salient given the aging of the U.S. population. The number of Americans aged 65 and older is projected to more than double from 46 million in 2016 to more than 98 million by 2060. The ≥65 age group will grow from 15% today to 24% by 2060.^59^

### Strengths and limitations

Symptom data were collected with a validated symptom instrument on arrival to the ED triage, eliminating most patient recall bias. A large heterogeneous sample was enrolled, suggesting that study findings may be generalizable to other EDs and patients with symptoms suggestive of ACS. Symptoms were self-reported by patients, which has been shown to be more reliable than symptoms abstracted from medical records.^60^ Self-report has also been considered to be a limitation because there is no way to validate the symptom event. Justice et al., however, noted that patients, not providers, experience symptoms and so are best able to describe them.^61^ We could not determine the inter-rater reliability of ECG analyses or whether the most recent AHA/ACC criteria (which account for sex and age differences in ST-segment deviations) were consistently used.^20^ Finally, we did not assess reproductive history and, hence, do not know the significance of pregnancy risks in our sample.

## Conclusions

Lack of chest symptoms and shortness of breath, key symptoms triggering a decision to seek emergent care, may influence older women's decision to delay treatment, placing them at risk for poorer outcomes. Younger African American women require more comprehensive risk reduction strategies and symptom management. Risk reduction strategies should target young women, particularly those who have had complications during pregnancy. Our findings reinforce the importance of tailoring ED assessment for potential ACS by age for women. Finally, the addition of nonchest pain symptoms in risk stratification models may be needed for older women.

## Abbreviations Used

ACC: American College of Cardiology
ACS: acute coronary syndrome
AHA: American Heart Association
CCI: Charlson Comorbidity Index
CI: confidence interval
ECG: electrocardiogram
ED: emergency department
MI: myocardial infarction
DASI: The Duke Activity Status Index
T2DM: type 2 diabetes
